# Development of a lab-device for evaporation-free supply of pure liquid nitrogen for droplet- and jet-generation

**DOI:** 10.1038/s41598-023-31955-4

**Published:** 2023-04-19

**Authors:** Markus Schremb, Marijn Kalter, Srinivas Vanapalli

**Affiliations:** Applied Thermal Sciences Lab; Energy, Materials and Systems cluster; Faculty of Science and Technology, University of Twente, Postbus 217, 7500 AE Enschede, The Netherlands

**Keywords:** Engineering, Fluid dynamics, Statistical physics, thermodynamics and nonlinear dynamics, Techniques and instrumentation

## Abstract

Cryogenic liquids such as liquid nitrogen are of relevance for numerous processes in engineering, and the food and pharmaceutical industries. However, as a result of its strong evaporation at ambient conditions, its handling for laboratory purposes and experimentation is so far cumbersome. In the present work an original design approach for a supply device for liquid nitrogen is developed and characterized in detail. With the device pure liquid nitrogen is supplied from a pressurized dewar flask to a hypodermic needle without contamination of the liquid with its own vapor or frost, finally enabling to generate a free liquid jet or single droplets in a way comparable to the handling of non-cryogenic liquids using a syringe and a hypodermic needle. Compared to previous approaches for the generation of liquid nitrogen droplets in scientific studies which mostly rely on a reservoir for liquid nitrogen from which droplets form at a bottom outlet due to gravity, the present design allows generation of droplets and free liquid jets in a significantly better controlled and more flexible way. The device is experimentally characterized for varying operational conditions during generation of a free liquid jet, and its versatility for laboratory research purposes is further briefly demonstrated.

## Introduction

Due to its importance for various processes in nature and engineering, droplet dynamics and more specifically droplet impact has been extensively studied experimentally for almost one and a half centuries^1,2^. For droplet generation even a syringe and an attached hypodermic needle may be sufficient in order to place a droplet wherever it is required; the droplet will simply detach from the needle due to gravity. The droplet size depends on the needle size and theoretically scales linearly with the needle diameter. If the needle is at the right position in the moment of detachment, that simple approach may be enough to perform droplet impact experiments. However, for more sophisticated purposes, e.g. for achieving a controlled droplet generation frequency or droplet size, or when handling non-Newtonian liquids, numerous design approaches for droplet generators have been reported in literature over the past decades^3–8^. These are either designed to fulfill a specific purpose in a certain industrial application or are used in a lab environment in order to study the fundamentals involved in a droplet’s interaction with its ambient. However, everything mentioned before solely relates to non-cryogenic liquids. Although a syringe and needle are basically sufficient for droplet generation, noticeable efforts have been invested in the design of droplet generators fulfilling the specific requirements resulting from a given application.

Based on the previous no further clarification is necessary to justify any efforts invested in the development of a system actually enabling droplet generation from a cryogenic liquid such as liquid nitrogen. Cryogenic liquids, i.e. liquids with boiling temperatures below − 150$\,^\circ$C at ambient pressure, are used in various fields such as engineering, and the food and pharmaceutical industries. Since cryogenic liquids permanently boil when being handled at ambient conditions, they exhibit a rather unique behavior. In particular the interaction of a cryogenic liquid with other liquids or solids being at higher temperatures is associated with various physical processes partially interacting with each other, and potentially controlling the technical process they are involved in. While their increasing exploitation for technical and medical applications basically motivates ongoing research with cryogenic liquids, continuous evaporation at ambient conditions tremendously complicates their handling for experimentation. It particularly prevents to use classical approaches for droplet generation which are well established for non-cryogenic liquids. Albeit commercial solutions do exist, until now only little is reported in literature about the generation of droplets from cryogenic liquids. However, specifically for liquid nitrogen used in scientific studies, droplets have so far mostly been generated by letting the liquid drip out of a stationary and insulated reservoir for liquid nitrogen^9–12^, which is associated with a significantly reduced versatility of the approach due to its limited flexibility and controllability of the liquid.

In the present work, an original design approach for a supply device for liquid nitrogen is presented and characterized. The device comprises a dewar flask for storage of liquid nitrogen and allows to supply small amounts of the liquid from the tip of a maneuverable hypodermic stainless steel needle at the end of a supply hose. The working principle of the device prevents contamination of the liquid with its own vapor and enables to continuously provide liquid nitrogen or to deposit small amounts of liquid nitrogen droplets from the needle tip. Using the cooling effect of sacrificial evaporation of a portion of the supplied liquid, evaporation inside the supply hose to the needle tip is prevented, finally allowing handling and use of liquid nitrogen comparable to handling a non-cryogenic liquid. Cryogenic fluids are commonly “sacrificed” for snap freezing in the scope of cryopreservation^13–15^, subcooling of stored cryogens^16^, or in order to maintain certain conditions during a laboratory experiment^17–19^. However, to the authors’ knowledge the approach and working principle developed in the present work have never been used before for a comparable purpose. The design approach and its working principle are presented in detail and the device functionality is characterized for the generation of a free liquid jet. In order to showcase its utility for research applications, the device versatility is briefly demonstrated based on high-speed video data showing the formation of a liquid droplet at the needle tip and the formation of a liquid nitrogen puddle on a warm substrate being in close proximity of the needle tip.

## The device

The device developed in the present work enables to provide pure liquid nitrogen without contamination with its own vapor at the tip of a small stainless steel hypodermic needle, which represents the end of a double-walled flexible tube connected to a dewar flask comprising the liquid. The working principle relies on the cooling effect of sacrificial evaporation of a portion of the liquid actually supplied through the inner part of the double-walled tube to the needle tip. A certain portion of the supplied liquid flows through small orifices in the end of the inner tube into the void volume of the outer tube where continuous sacrificial evaporation of that liquid prevents evaporation of the liquid actually transferred through the inner tube. Compared to previous approaches for droplet generation from liquid nitrogen which are all accompanied by a certain unhandiness, the needle at the end of the flexible tube in the present approach is hand-held and maneuverable, and thus, allows to generate and place droplets and jets in a much more flexible way. The device design, its equipment with measurement technique for temperature, pressure and efficiency, as well as its general operation principle are separately described in the following sections.

### Device design

**Figure 1:**
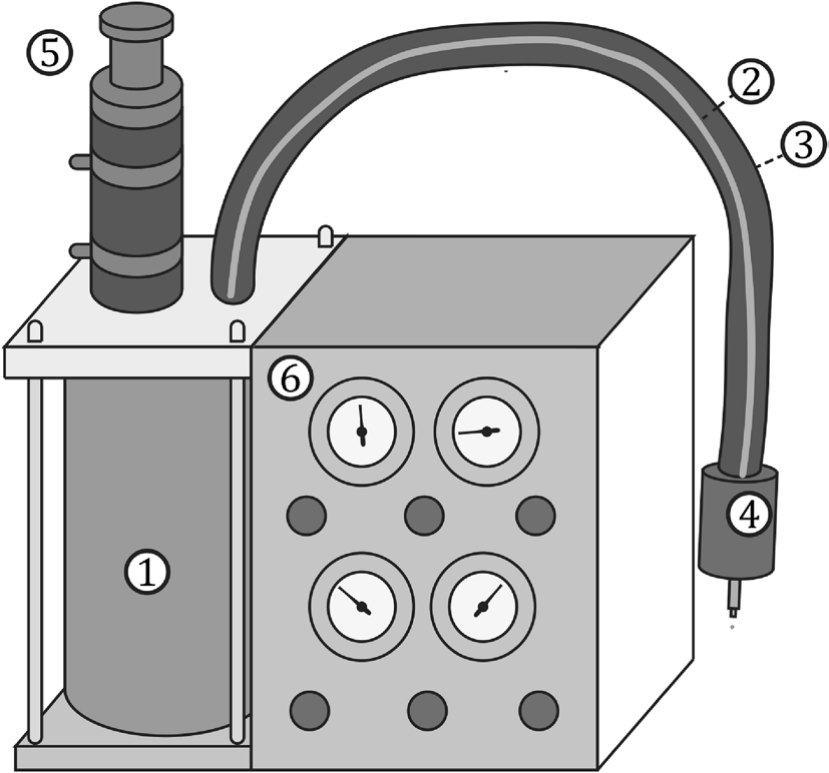
Schematic illustration of the nitrogen supply device comprising the dewar flask (1), the inner (2) and outer (3) tube, the handpiece (4), a safety valve (5) and the front panel (6) with valves and gauges for controlling and monitoring the device behavior. Foam insulation actually placed around the double-walled tube is not shown in the picture.

The device consists of a stationary case, a handpiece which is maneuverable in order to dispense droplets and direct liquid jets wherever they are required, and a double-walled tube of approximately $1.2\,\hbox {m}$ length connecting these two main parts, as schematically shown in Fig. 1. The length of the connecting tube may be theoretically varied. However, a significant extension of its length presumably also requires adaptations of the remaining system in order to compensate for the increased heat transfer from the ambient to the tube.

The stationary part of the device comprises a vacuum-insulated dewar flask with the cryogenic liquid inside, and is connected to both a source for pressurized gaseous nitrogen and a vacuum pump (*Anest Iwata, DVSL-100C*). The device operation is coarsely regulated and monitored through control valves and analog pressure gauges placed in the case front panel. As an indication for the dimensions of the device, the height of the front panel is approximately $270\,\hbox {mm}$. Note that for the purpose of clarity not all device components are shown in the schematic in Fig. 1. The dewar flask with a volume of approximately 2 liters is closed gas-tight with a transparent PMMA lid and a flexible rubber mat for sealing. The lid is equipped with different feed-throughs and allows to optically monitor the dewar filling status. In addition to the natural pressure build-up due to liquid evaporation, the dewar can be actively pressurized from the source of pressurized gaseous nitrogen. While a mass flow controller (*Bronkhorst, EL-FLOW Select*) at a vent connection on the dewar lid is used to control the dewar pressure to a constant value, an overpressure safety valve ultimately limits the absolute pressure inside the dewar to approximately 1.9 bar.

**Figure 2:**
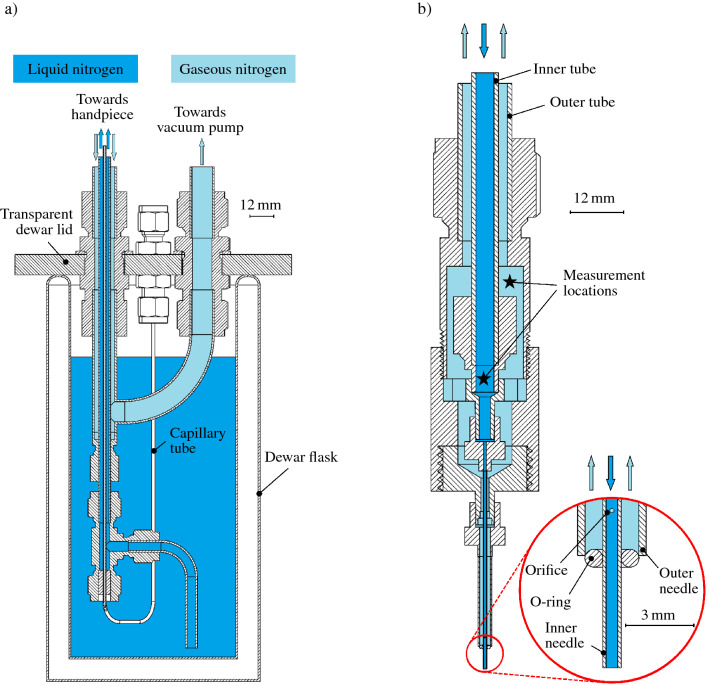
In-scale cross-sectional views of (**a**) the dewar flask with the tube connections for liquid and gaseous nitrogen inside and through its lid, and b) the device handpiece connecting the plain ends of the inner and outer flexible tube (top) with the inner and outer hypodermic needle (bottom), respectively. Arrows in the figures indicate the flow direction of liquid (dark blue) and mainly gaseous (light blue) nitrogen in the system. The lid connection used to pressurize the dewar and a filter element at the inner tube inlet are not shown in (**a**). Stars in (**b**) indicate the measurement locations for pressure and temperature in the liquid and gaseous domain inside the handpiece. The situation around the orifices in the inner needle tip is shown in a detailed view in (**b**).

A flexible corrugated stainless steel tube with an outer diameter (O.D.) of 6 mm and an effective inner diameter (I.D.) of 3.8 mm is used as the liquid nitrogen tube connecting the dewar with the device handpiece. It is referred to as the *inner tube* in the following. The tube is installed in the dewar basically in the form of a dip tube, as shown in Fig. 2a). Therefore, pressurizing the dewar results in a flow of nitrogen towards the handpiece. A sintered plastic silencer commonly used for pneumatics (not shown in Fig. 2) is attached to the end of the inner tube inside the dewar and is used to filter the liquid nitrogen entering the inner tube, thus preventing contamination of the system. Along its entire length from the dewar to the handpiece, the inner tube is surrounded by a larger flexible corrugated stainless steel tube with effective inner and outer diameters of approximately $12\,\hbox {mm}$ and $16\,\hbox {mm}$, respectively. This tube is referred to as the *outer tube* or *gaseous transfer line* in the following. Plain stainless steel tubes are soldered to the ends of the inner and outer steel tube and all connections of these ends are realized using cutting ring fittings, enabling leak-tight sealing and re-usable (dis-)assembly of the system.

The outer tube is fed through the dewar lid while still surrounding the inner tube, as shown in Fig. 2a). It is sealed around the inner tube inside the dewar and the void volume in between these tubes, i.e. the gaseous transfer line, is connected to a feed-through in the dewar lid, which ultimately connects towards the vacuum pump. A needle valve installed in the connection between this feed-through and the vacuum pump allows adjustment of the flow rate and thus, control of the resulting pressure in the gaseous transfer line. At the other end of the double-walled tube, the inner and outer tube are attached to the handpiece, as shown in Fig. 2b). Via the handpiece the inner and outer tube are connected to hypodermic stainless steel needles (*Hamilton Company*) of 21 needle gauge ($0.51\,\hbox {mm}$ I.D., $0.82\,\hbox {mm}$ O.D.) and 11 needle gauge ($2.39\,\hbox {mm}$ I.D., $3.05\,\hbox {mm}$ O.D.), respectively. Similar to the situation of the two flexible tubes, also the larger needle surrounds the smaller needle, and both represent the miniaturized ends of the outer and inner tube, respectively. During device operation, liquid nitrogen is provided through the inner needle in order to generate a continuous liquid jet or a single droplet at the needle outlet. An O-ring is used to seal the end of the outer needle around the inner needle which sticks out over the outer needle by approximately $5\,\hbox {mm}$, as shown in the detail in Fig. 2b). In order to fix the O-ring to that position, two pieces of flexible rubber tube are imposed on the needles (not shown in the figure for the purpose of clarity).

Two small orifices with a diameter of approximately $0.2\,\hbox {mm}$ are drilled into the inner needle at a distance of $7\,\hbox {mm}$ from the needle tip, as shown in the detail in Fig. 2b). The orifices connect the inside of the inner needle with the void volume of the outer needle which actually ends at the vacuum pump connected to the feed-through in the dewar lid, as indicated in Fig. 2a). Rubber tube insulation around the outer tube minimizes heat transfer from the ambient to the tube and frost formation on the tube. Note that at room temperature, the double-walled tube including its insulation is highly flexible. However, during device operation, i.e. when the tube is at typical operation temperatures ($\approx 77$ K), the insulation material around it gets relatively brittle allowing only little re-positioning of the tube after device startup.

### Device operation

During operation of the device the inner tube is completely filled with liquid nitrogen. The minimum pressure of the liquid is at the very end of the inner needle where it equals ambient pressure. At all other positions in the inner tube, the liquid pressure is above ambient pressure and thus, also the saturation temperature of the liquid is above the saturation temperature at ambient pressure at every position. However, due to the small flow rate of liquid nitrogen ($\mathscr {O}(1\,\hbox {ml}/\hbox {s})$) through the system and particularly through the comparably large inner tube, the pressure loss in the flow from the dewar to the needle tip can be assumed to be negligibly small. Consequently, also the variation of the saturation temperature along the tube can be neglected. For nitrogen being in thermal equilibrium inside the dewar during device operation, the liquid enters the inner tube with a temperature above the saturation temperature at ambient pressure, namely with the saturation temperature associated with the increased pressure inside the dewar.

Decreasing the pressure in the outer tube with respect to ambient pressure results in a certain flow of liquid nitrogen from the inner needle through the orifices into the void volume of the outer needle and outer tube. Due to the decreased pressure, the saturation temperature in the outer tube is lowered compared to the saturation temperature at ambient pressure and thus, it is also below the saturation temperature of the liquid at every position inside the inner tube. Liquid nitrogen soaked through the orifice into the outer tube evaporates at that lower saturation temperature, while both the ambient and the liquid in the inner needle and inner tube are at higher temperatures. Therefore, evaporation of the liquid in the outer tube actually serves as a heat sink capable of dissipating the heat originating from heat transfer from the ambient and heat transfer from the warmer inner tube. For a sufficient inflow and subsequent evaporation of sacrificial liquid at decreased pressure in the outer tube, liquid evaporation in the inner tube is prevented along the entire tube length, finally enabling to provide pure liquid nitrogen from the dewar to the inner needle tip. Note that although the outer tube is also referred to as the gaseous transfer line, at least over a certain distance from the orifices a multiphase mixture of evaporating liquid and its vapor flows through it.

Although the device operation is quite complex, the previous explanation is sufficient for a basic understanding of the results presented in the present work. However, a detailed explanation of the device operation during idle, for jet- and droplet- generation, as well as during device start-up is provided in the supplementary material to this manuscript.

### Measurement equipment

Due to the small dimensions of the hypodermic needles, measurements of temperature and pressure directly before and after the orifices in the inner needle are not possible. Instead, the measurements are performed as close as possible to the orifices, as indicated with stars in Fig. 2b). Although the measured data may not be perfectly representative for the actual situation before and after the orifices, it is the most representative data available to characterize device operation and the situation around the orifices. A stainless steel capillary tube ($0.75\,\hbox {mm}$ I.D., $1.6\,\hbox {mm}$ O.D.) for pressure measurement in the liquid inside the handpiece is inserted into the inner tube inside the dewar, as shown in Fig. 2a). Similarly, a thermocouple is inserted into the inner tube inside the dewar ending at the measurement location indicated in Fig. 2b). For the temperature and pressure measurements in the gas inside the handpiece, a feed-through into the plain end of the outer tube allows insertion of a thermocouple and a capillary tube, respectively. The temperature in the liquid transfer line is also measured half-way between the dewar and the handpiece. However, the measurements at that location are not used for device characterization in the present study, but provide a good indication for the current state of the device during its start-up. The temperature of the free liquid jet is not directly measured in the experiments. However, the temperature data for the liquid inside the hand piece may serve as a good estimate for that temperature. More specifically, the measured temperature represents the upper bound for the temperature of the liquid at the needle exit. Starting at the measurement position inside the hand piece, the liquid flow through the inner needle will further experience cooling from evaporation inside the outer needle, such that the liquid finally leaves the inner needle with an even lower temperature compared to the liquid temperature measured before entering the inner needle. However, its exact value is not measured in the experiments.

The measurements are performed using two-point calibrated Type E thermocouples (*Omega, PFA insulated, 36AWG*) and an USB thermocouple data logger (*Picotech, TC-08*) with a final accuracy of approximately $\pm 0.4\,\hbox {K}$, and pressure sensors (*GE, Unik 5000 series*) with an accuracy of approximately $\pm 16$ mbar connected to the capillary tubes. A mass flow meter (*Bronkhorst, EL-FLOW Select*) is used to measure the mass flow rate of gaseous nitrogen through the outer tube towards the vacuum pump with an accuracy of approximately $\pm 12$ mg/s. All data is sampled with a rate of approximately 1 Hz using a data acquisition device (*National Instruments, USB 6218*). While the relatively low sampling rate does not allow to resolve the evolution of the measured quantities during transient operation, it is fully sufficient for the majority of measurements performed during quasi-steady device operation.

All measurements for quantitative device characterization during continuous operation are performed with a horizontally ejected liquid nitrogen jet and the situation at the needle exit is captured in a side view using a high-speed video camera (*Photron, FASTCAM NOVA S6*) and backlight illumination. The camera is operated with a minimum spatial resolution of approximately $19\,\upmu \hbox {m}/\hbox {pixel}$ and depending on the given case with a frame-rate between 50 and 1000 fps. In order to reduce frost formation at the cold needle tip, the needle is inserted into a transparent plastic box which is continuously flushed with gaseous nitrogen. The flow velocity of the liquid jet at the needle outlet is measured from the captured video data using in-house video-postprocessing codes implemented in the commercial software package Matlab (*The Mathworks*). Neglecting viscous forces acting on the jet in its longitudinal direction and actually decelerating it, the jet outlet velocity is determined from fitting the equation for a ballistic trajectory to the skeleton line of the liquid jet and the center of mass of the individual droplets originating from the jet. The mass flow rate through the needle tip is determined assuming a circular liquid jet with a diameter equal to the inner diameter of the inner needle, and using the density of liquid nitrogen at ambient conditions, $808.22\,\hbox {kg}/\hbox {m}^3$^20^. Note that neglecting the decelerating force acting on the liquid jet results in an underestimation of the jet velocity at the needle outlet measured from the video data.

For continuous device operation, the measurements of the mass flow rate through the outer tube and the optical measurement of the outflow velocity of the liquid jet are used to calculate the efficiency of the device. It compares the resulting mass flow of liquid nitrogen at the needle outlet with the total mass flow of liquid nitrogen consumed for device operation as

1 $$\begin{aligned} \eta =\frac{\dot{m}_\mathrm {jet}}{\dot{m}_\mathrm {tot}}, \end{aligned}$$

where $\dot{m}_\mathrm {jet}$ and $\dot{m}_\mathrm {tot}$ denote the mass flow rate through the ejected jet and the total mass flow rate of liquid nitrogen from the dewar, being the sum of the mass flow of the liquid jet and the measured mass flow of nitrogen gas to the vacuum pump, $\dot{m}_\mathrm {gas}$. Note that due to the underestimation of the jet velocity from the optical measurement, the described method for calculating the device efficiency only provides a conservative estimate; the actual device efficiency is higher.

## Results

The device functionality is mainly examined and quantified for the continuous operation mode, i.e. for the generation of a free liquid jet. However, the device versatility is also briefly demonstrated based on high-speed video data showing the formation of a droplet growing at the needle tip, and a liquid puddle developing on a warm brass substrate placed close below the needle tip. While the free liquid jet is ejected horizontally during continuous operation in order to allow optical jet velocity measurement, the hand piece and needle are placed vertically for droplet and puddle formation.

### Jet generation

Jet generation is examined both qualitatively and quantitatively. While high-speed video data showing the outflow from the needle tip is used for a qualitative demonstration of the device function, the effect of varying operational conditions on the resulting temperatures measured inside the handpiece, the liquid jet velocity and the device efficiency is quantitatively examined.

#### Qualitative demonstration

**Figure 3:**
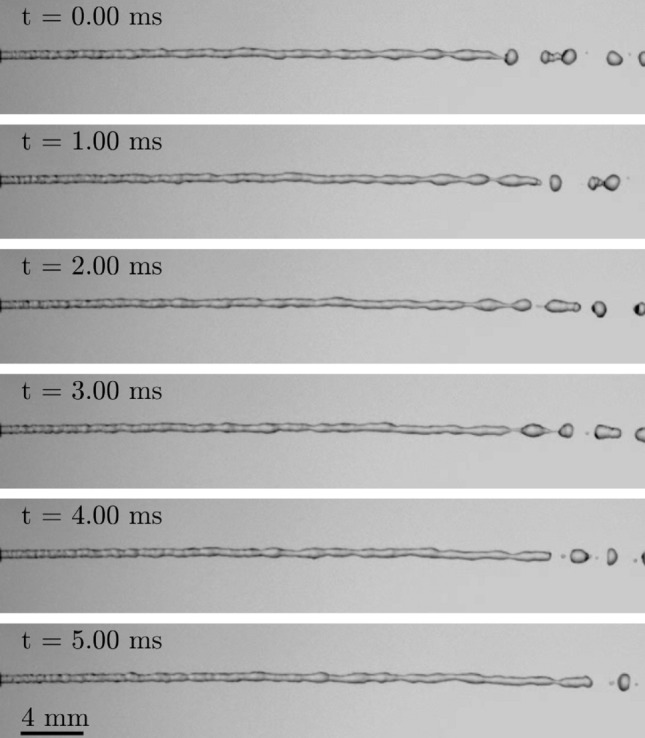
Example free liquid nitrogen jet formed at the needle outlet during device operation with $p_l \approx 1.6$ bar and $p_g \approx 0.8$ bar. The liquid leaves the needle with approximately $v_{jet}=2.97$ m/s resulting in a mass flow of approximately $\dot{m}=0.47$ g/s and a device efficiency of $\eta \approx 60\%$.

A free liquid nitrogen jet resulting from device operation with liquid and gaseous pressures of $p_l \approx 1.6$ bar and $p_g \approx 0.8$ bar, respectively, is shown as an example in Fig. 3. For these conditions, the liquid leaves the needle with approximately 2.97 m/s which results in a jet mass flow of approximately 0.47 g/s and a device efficiency of $\eta \approx 60\,\%$; i.e. 40 % of the consumed liquid nitrogen are required for device operation and do not contribute to the liquid flow leaving the needle tip. As shown in the figure, the appearance of the nitrogen jet is absolutely similar to that of a free jet of a non-cryogenic liquid. The liquid leaves the needle as a continuous jet with a rather smooth and only slightly disturbed surface. However, the Rayleigh-Plateau instability of the jet surface causes irregularities of the jet contour to grow and finally results in jet break-up into individual droplets.

**Figure 4:**
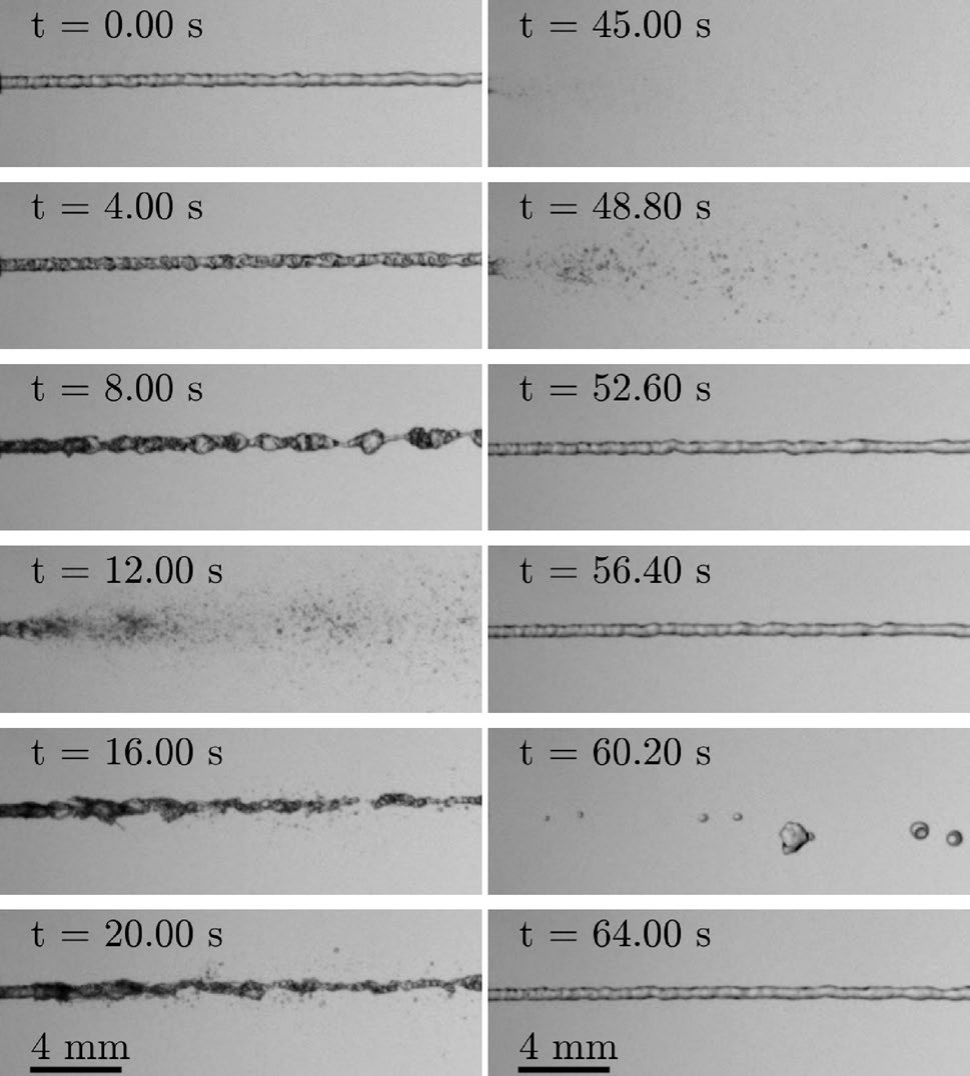
Temporal evolution of the outflow from the needle tip resulting from interrupting the connection between the outer tube and the vacuum pump at $t=0$, and after re-establishing the connection at $t=45\,\hbox {s}$. Before interruption, the device operated in equilibrium at $p_l \approx 1.6$ bar and $p_g \approx 0.8$ bar corresponding to the operation shown in Fig. 3. The shown data during shut-off and re-start of the method correspond to the measurements shown in Fig. 5.

**Figure 5:**
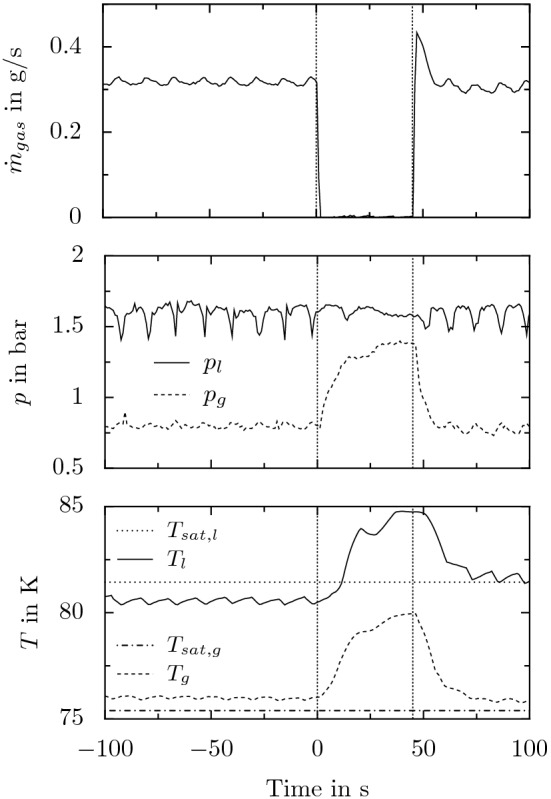
Temporal evolution of the mass flow to the vacuum pump, and the pressures and resulting temperatures in the liquid and gaseous domain inside the device handpiece during temporary disconnection of the vacuum pump from the outer tube starting at $t=0$. At time $t=45\,\hbox {s}$ the connection is re-established. The data for $t<0$ and $t \ge 0$ corresponds to the situation at the needle outlet, shown in Figs. 3  & 4, respectively. The dotted and dash-dotted horizontal lines in the lowest figure correspond to the saturation temperature related to the average pressures during stable operation, $p_l$ and $p_g$, respectively^20^.

The effect of temporarily stopping sacrificial evaporation, i.e. interrupting the connection between the vacuum pump and the outer tube, on the outflow from the needle after stable operation with $p_l \approx 1.6$ bar and $p_g \approx 0.8$ bar is shown in the left column of Fig. 4; the result of again re-establishing the connection is shown in the right column of the figure. Time $t=0$ refers to the moment of interrupting the connection to the vacuum pump, which is re-established at $t \approx 45\,\hbox {s}$. The corresponding temporal evolution of the mass flow rate towards the vacuum pump and the resulting pressures and temperatures measured in the liquid and gaseous domain inside the handpiece is shown in Fig. 5. While the pressure data represents the raw measurement data as it is sampled, the shown temperature data is the result of a moving average filter with a window width of approximately 10 seconds applied to the measurement data. The dashed vertical lines in the figure mark the moment of interrupting and re-establishing the connection to the vacuum pump at $t=0$ and $t=45\,\hbox {s}$, respectively. The horizontal dotted and dash-dotted line in the graph for the temperature data represents the saturation temperatures corresponding to the pressures in the handpiece measured before disconnection of the vacuum pump, i.e. to $p_l \approx 1.6$ bar and $p_g \approx 0.8$ bar, respectively. It is worth to note that the data for the mass flow rate does not reflect the mass flow rate of expanding liquid nitrogen during transient operation. Therefore, the increased mass flow rate measured after re-connection of the vacuum pump does not necessarily reflect an increased rate of nitrogen evaporation. Its increase is rather a result of the fluid pressure and density in the outer tube which increased during disconnection of the pump.

The data for stable operation ($t<0$) in Fig. 5 actually corresponds to the situation at the needle outlet shown in Fig. 3. As shown in the figure, during stable operation all measured quantities are associated with a certain fluctuation with a typical frequency in the order of $\mathscr {O}(0.01)\,\hbox {Hz}$, whose origin is unfortunately not completely clear. Actually, both constant average conditions and a stable liquid jet leaving the needle outlet indicate operation of the device in the desired way, i.e. with purely liquid nitrogen inside the inner tube. However, particularly the fluctuation of the liquid pressure inside the hand piece is most probably attributed to the occurrence of phase change and its effect on the fluid pressure. While the fluctuations of $p_g$ are much less pronounced and most probably only a result of the fluctuations of $p_l$, local effects in the liquid flow in the hand piece may indeed cause phase change which may affect the pressure measurements, even when the average flow conditions theoretically correspond to a stable liquid phase. The liquid temperature and pressure inside the needle head are measured locally at the tip of a thermocouple and a capillary tube, respectively. Consequently, the measured data only reflects the local conditions at the measurement position and does not necessarily well represent the entire flow situation. Similar as in the case of cavitation for non-cryogenic fluid systems^21^, the fluid pressure may be locally reduced below the fluid’s saturation pressure, finally causing evaporation and bubble formation inside the liquid, which in turn affects both the local pressure and temperature. However, when further travelling downstream with the surrounding flow the bubbles again collapse in regions of elevated pressure. While cavitation usually concerns systems with liquid pressures well above the saturation pressure for the given fluid temperature, the situation in the present case is even worse. The close vicinity between the fluid pressure and its saturation pressure presumably increase the chance for cavitation in the present fluid system. While such local phenomena apparently do not disturb the macroscopic device behavior in terms of the average operating conditions and the resulting jet at the needle outlet, they may be the reason for the huge local fluctuations as observed in the measurements. The characteristic frequency associated with the described effects is typically much higher than the observed oscillation frequency of $\mathscr {O}(0.01)\,\hbox {Hz}$. Nevertheless, the described mechanism cannot be completely ruled out to contribute to the observed oscillatory behavior. However, the actual mechanisms relating to the observed oscillation frequency can not be clearly identified at the moment.

As shown for stable operation ($t<0$) in Fig. 5, the liquid temperature is continuously below the saturation temperature at the given liquid pressure. However, the temperature in the gaseous domain is slightly above the saturation temperature corresponding to the given pressure, which presumably is due to complete evaporation of the liquid being soaked into the outer tube before reaching the measurement location for temperature and pressure. Heat from the inner tube and from ambient is not only dissipated through sacrificial evaporation of the liquid at reduced pressure but also through an increase of sensible heat of the resulting colder nitrogen gas in the outer tube. This hypothesis has actually been confirmed through different tests for which the device was equipped with a needle with slightly larger orifices. These tests revealed an almost perfect agreement (approximately $0.1\,\hbox {K}$ difference) between the resulting temperature in the gaseous domain and the saturation temperature corresponding to the given pressure. In the case of a larger orifice size, for given pressure conditions more liquid is soaked into the outer tube, while the heat to be dissipated from the inner tube and from ambient is unaffected from the orifice size. As a result, sacrificial evaporation of the liquid may serve to dissipate more heat and less heat needs to be dissipated through a change of the sensible heat of the resulting nitrogen gas. Consequently, for an increasing mass flow of liquid nitrogen into the outer tube as a result of an increasing orifice size, $T_g$ approaches saturation temperature. Although the entire liquid seemingly evaporates before reaching the measurement location for pressure and temperature, a certain amount of liquid nitrogen may still be present between the measurement location and the orifices.

After interruption of the connection between the vacuum pump and the outer tube, the thermal inertia of the device components prevents immediate collapse of the thermal equilibrium and the resulting pure liquid jet, as shown in Fig. 5. While the temperature in the gas domain, $T_g$, starts to increase almost simultaneously with the increasing pressure, the liquid temperature, $T_l$, starts increasing just after a certain delay. Approximately $3.6\,\hbox {s}$ after interruption, first bubbles in the jet become visible and for $t=4\,\hbox {s}$ a homogeneous mixture of liquid nitrogen and immersed vapor bubbles leaves the needle. Due to further warm up of the components to a temperature well above saturation, the portion of nitrogen gas leaving the needle continuously increases while that of the liquid decreases, finally resulting in a highly chaotic multiphase jet at the needle outlet. As shown for $t=12\,\hbox {s}$, the high portion of gas in the stream may even result in atomization of the liquid to a fine spray of nitrogen droplets. However, neither that spray nor the chaotic multiphase jet present at later times are stable and instead, the situation at the needle outlet stochastically alternates between these two states during the observed time span. Due to a continuing warm-up of the device, the situation for later times more and more tends to the atomized or even purely gaseous outflow from the needle such as shown for $t=45\,\hbox {s}$.

After re-establishing the connection between the vacuum pump and the outer tube at $t \approx 45\,\hbox {s}$, the cooling effect of sacrificial evaporation of liquid nitrogen in the outer tube is re-established. As a result, starting with an almost purely gaseous outflow at the needle tip, evaporation in the inner tube is increasingly suppressed until a pure liquid nitrogen jet leaves the needle again $10\,\hbox {s}$ after re-connection of the vacuum pump. However, at that moment the device operation is not yet completely back in equilibrium and the temperatures are still higher than during stable operation at $p_l \approx 1.6$ bar and $p_g \approx 0.8$ bar as shown in Fig. 5. Consequently, evaporation in the inner tube may still result in an explosive and discontinuous outflow from the needle as shown for example for $t=60.2$ s in Fig. 4. However, for later times, $t>64$ s, the outflow from the needle is stable and purely liquid again. At that time, the temperature in the gas domain is back again at the value present before disconnecting the vacuum pump, while the temperature in the liquid domain is still well above the initial temperature and continues to decrease. Although the liquid temperature is still slightly above the saturation temperature for the given pressure in the liquid domain, evaporation in the inner tube is already completely suppressed allowing supply of pure liquid nitrogen from the needle again.

As shown for the present example, the device may be brought back to stable operation again after a collapse of the thermal equilibrium and a certain warm-up of the device components. However, the time of interruption of sacrificial evaporation is limited and after a too long interruption and the accompanied warm-up, the thermal equilibrium can not be re-established again. In that case, the amount of liquid nitrogen reaching the orifices is finally too small to cool-down the device components again, which has to be accomplished according to the common start-up procedure explained in the supplementary material of the present manuscript.

#### Quantitative characterization

**Figure 6:**
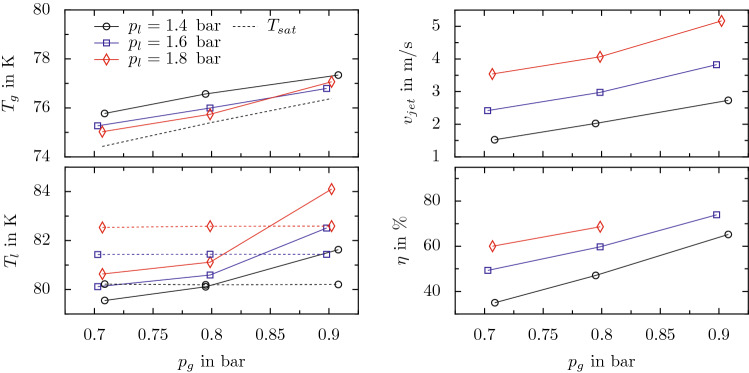
Effect of varying operation pressures $p_g$ and $p_l$ on the resulting temperatures $T_g$ and $T_l$, the jet velocity $v_{jet}$ and the device efficiency $\eta$. Dashed lines in the graphs for temperature represent the saturation temperature for the respective pressures. In the graph for $T_g$, the saturation temperature refers to $p_g$, while it corresponds to $p_l$ in the graph for $T_l$.

For a quantitative characterization of the device function, the operating pressures $p_g$ and $p_l$ have been independently varied between approximately 0.7 bar and 0.9 bar, and 1.4 bar and 1.8 bar, respectively. The effect of these variations on the resulting averaged values of the temperatures $T_g$ and $T_l$, the jet velocity $v_{jet}$ and the device efficiency $\eta$ is shown in Fig. 6. Dotted lines in the graphs for $T_g$ and $T_l$ represent the saturation temperature corresponding to the pressures in the gaseous and liquid domain, respectively. The device efficiency $\eta$ is not provided for $p_g=0.9$ bar and $p_l=1.8$ bar, since for that case sacrificial evaporation is not sufficient to prevent evaporation in the inner tube, finally resulting in the outflow of a multiphase-jet from the needle. Therefore, the exact composition of the resulting jet is not known and thus, the jet mass flow and the derived device efficiency cannot be determined. However, the jet velocity and temperatures are unaffected from the jet composition and thus, are provided also for these conditions.

As expected and shown in Fig. 6, an increasing pressure in the gaseous domain, $p_g$, i.e. decreasing cooling from sacrificial evaporation in the outer tube, results in both a higher $T_g$ and $T_l$. Moreover, it is associated with an increasing jet velocity and device efficiency, which both depend nearly linearly on $p_g$. Apart from the data for $T_g$ at $p_g=0.9$ bar, for which sacrificial evaporation is not sufficient and the device does not provide a pure liquid jet, all data shows a clear trend for both varying liquid pressure, $p_l$, and varying gas pressure, $p_g$. The higher $p_l$, the lower is the temperature in the gas domain, $T_g$, and the higher are the temperature in the liquid domain, $T_l$, the jet velocity $v_{jet}$ and the device efficiency, $\eta$. As already shown before for $p_l \approx 1.6$ bar and $p_g \approx 0.8$ bar in Fig. 5, due to complete evaporation of the liquid in the outer needle before it reaches the measurement location for temperature in the hand piece, $T_g$ is always above saturation. However, its excess above saturation decreases for increasing $p_l$. Interestingly, the relation between the actual temperature and the saturation temperature in the liquid domain varies for varying $p_g$, as seen in the lower left graph. While $T_l$ is below the corresponding saturation temperature in the liquid domain for all cases with $p_g \le 0.8$ bar, the temperature in the liquid domain is consistently above the corresponding saturation temperature for $p_g=0.9$ bar, which should theoretically cause liquid evaporation in the inner tube. While this clearly results in a multiphase jet for the situation with $p_g=0.9$ bar and $p_l=1.8$ bar which has already been mentioned before, no gas contamination of the jet is observed for smaller liquid pressures $p_l<1.8$ bar at $p_g=0.9$ bar; even though also for these conditions the liquid temperatures are well above the corresponding saturation temperature. Presumably, the nucleation barrier for bubble formation may cause the liquid jet to be still free of gas contamination even though its temperature is above saturation. However, a definite explanation can not be provided at the moment. For smaller pressures in the gas domain, $p_g \le 0.8$ bar, the “thermal buffer”, i.e. the undershoot of the actual liquid temperature below the saturation temperature increases with increasing $p_l$. For a constant pressure $p_g=0.7$ bar, the thermal buffer is only 0.7 K for $p_l=1.4$ bar while it amounts to even 1.9 K for $p_l=1.8$ bar. Concluding from these results the most stable conditions for device operation correspond to a preferably large $p_l$ in conjunction with a preferably small $p_g$.

**Figure 7:**
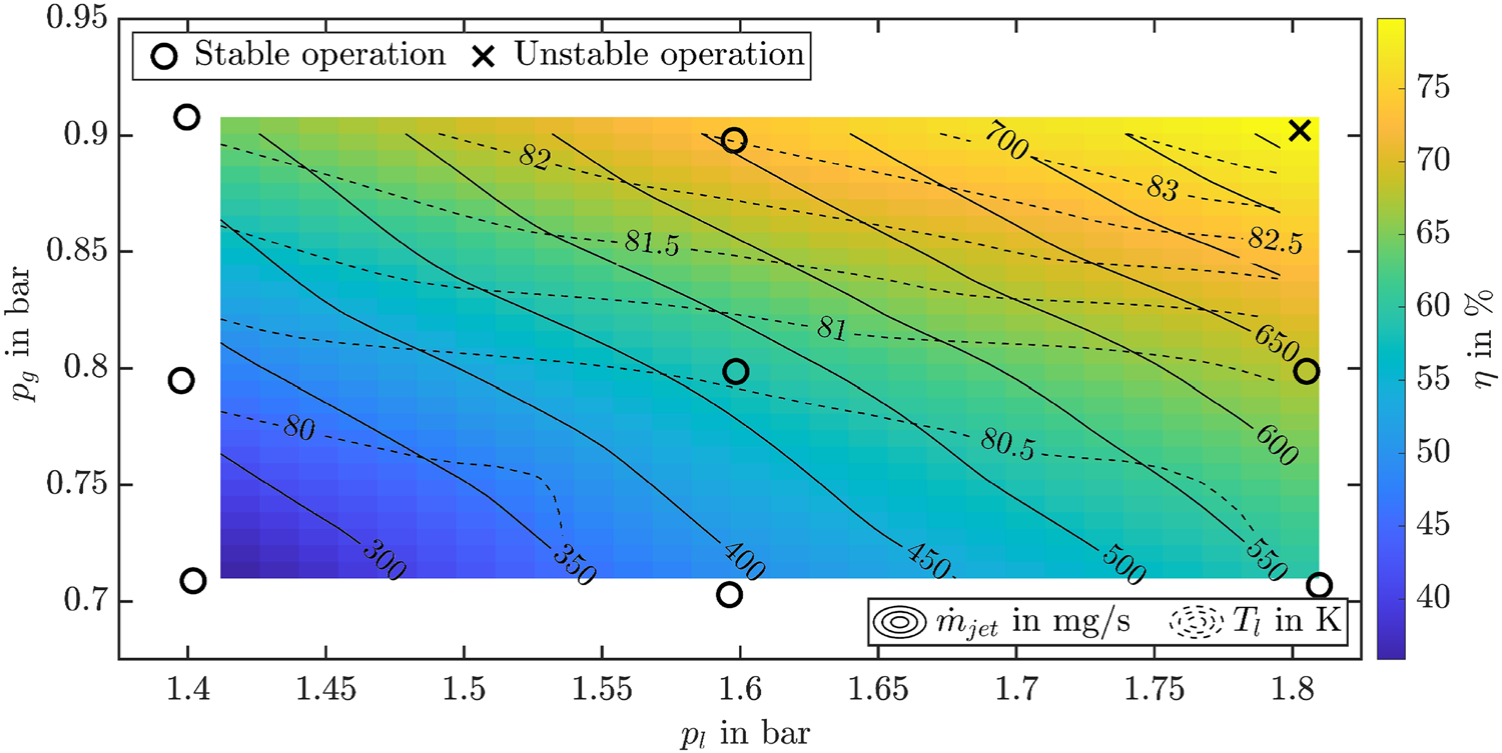
Operational diagram summarizing the conditions during device operation resulting from varying combinations of the pressures $p_l$ and $p_g$. The pressure combinations used in the experiments are indicated in the diagram using symbols, where circles and crosses refer to stable and unstable operation, respectively. The data for $\dot{m}_{jet}$ (solid lines) and $T_l$ (dashed line) as well as for $\eta$ (color) is derived from cubic interpolation of the experimental results.

The resulting conditions in terms of $\dot{m}_{jet}$, $T_l$ and $\eta$ associated with device operation at varying pressures $p_l$ and $p_g$ are summarized in an operational diagram in Fig. 7. The device behavior observed for the tested conditions is indicated in the diagram using symbols, where circles refer to stable device operation with pure liquid nitrogen at the needle outlet and the cross indicates unstable device behavior with significant gas inclusions in the liquid jet. Based on the present experimental results, the contour lines for $\dot{m}_{jet}$ (solid lines) and $T_l$ (dashed line), and the field for $\eta$ are obtained from cubic interpolation. Due to the limited data base from the present experiments, the accuracy of the data in the diagram is also limited. Therefore, the evolution of the contour lines for $T_l<81$ K at comparably high $p_l$ is presumably not physical, but rather related to the limitations for interpolation based on the present data base. Nevertheless, the operational diagram provides a good overview and first a-priori estimate of possible operational conditions of the device. For example, the diagram allows to determine the pressure conditions required to establish certain combinations of the jet mass flow rate, $\dot{m}_{jet}$, and the liquid temperature $T_l$. Even though the resulting conditions may not be accurately predicted, the data at least gives a good estimate of the possibilities in the given range of pressures.

### Further applications

In addition to the previous device characterization for the continuous operation mode, the device versatility is briefly demonstrated based on high-speed video data showing the formation process of a droplet detaching from the needle tip and of a needle-bound liquid puddle generated on a warm brass substrate. Both examples are of relevance for fundamental studies using liquid nitrogen or cryogenic liquids in general. Employing the device for droplet generation for example allows examination of dynamic droplet impact or other forms of droplet deposition, while the ability for controlled liquid puddle formation for example enables detailed examination of the Leidenfrost state of liquid nitrogen above the substrate below, such as reported in reference^22^ for a non-cryogenic liquid.

Equilibrium operation of the device is the starting point for droplet and puddle formation. In that case the operational conditions are such that the mass flow of liquid nitrogen in the inner tube equals the mass flow to the vacuum pump in the outer tube. Consequently, the inner tube is filled with liquid nitrogen but theoretically no liquid leaves the needle outlet. As explained more in detail in the supplementary information, the equilibrium operation point is rather sensitive to operational changes and moreover, it is associated with a certain oscillation of the resulting cooling effect through sacrificial evaporation, as already discussed in the scope of Figs. 3 and 5. Therefore, adjustment of the conditions for equilibrium operation is non-trivial and may require several trials for finding proper settings. However, in comparison to current approaches for droplet generation from liquid nitrogen or for handling of small portions of liquid nitrogen for lab-purposes, the developed device is superior already in its present state. Any further improvements and optimizations of the device are actually out of the scope of the present work and should be rather part of future development.

#### Droplet generation

**Figure 8:**
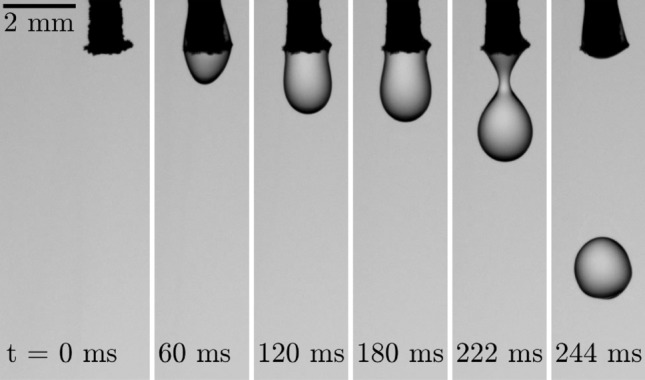
Droplet formation at the needle outlet for the device operated in equilibrium at $p_l \approx 1.15$ bar and $p_g \approx 0.45$ bar. Time $t=0$ refers to the moment, when the first liquid is visible to leave the needle after over-pressurizing the outer tube for approximately 50 ms.

The formation of a liquid nitrogen droplet at the needle tip is shown as an example in Fig. 8. It is the result of temporarily pressurizing the outer tube with an over-pressure of approximately 1 bar for approximately 50 ms during equilibrium operation at $p_l \approx 1.15$ bar and $p_g \approx 0.45$ bar. Instead of an impulsive shoot-out of liquid from the needle such as in the case of common drop-on-demand droplet generators for non-cryogenic liquids, the liquid rather flows out of the inner needle due to a temporary excess mass flow of liquid nitrogen as a result of the temporarily reduced mass flow through the outer tube. The relative slowness of that process is also indicated by liquid nitrogen partially wetting the outside of the needle tip, visible for $t=60$ ms in the figure.

While the liquid pressure is relatively small in the present example, an increased liquid pressure and thus increased flow rate of liquid through the inner tube is expected to reduce oscillatory behavior during idle operation. However, for the case of an increased mass flow through the inner tube, the small orifices used in the present study do not allow the mass flow into the outer tube which is required for idle operation. Therefore, further improvement of the device in particular for idle operation for droplet generation requires further detailed examination of the device including a variation of the orifice size and operation pressures during idle.

#### Puddle formation

**Figure 9:**
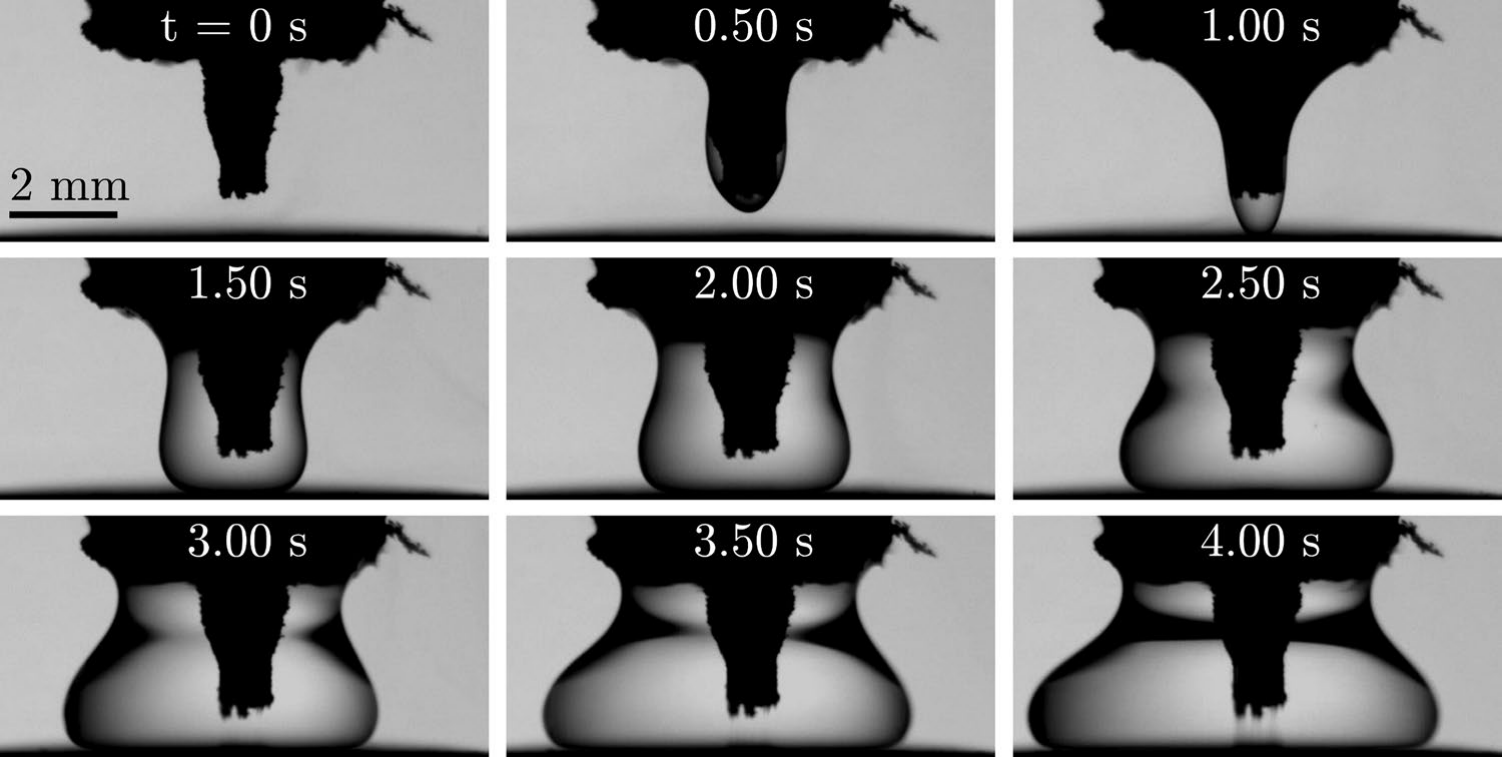
Growth of a needle-bound liquid nitrogen puddle on a warm brass substrate after equilibrium operation of the device at $p_l \approx 1.1$ bar and $p_g \approx 0.5$ bar. Starting from equilibrium operation, the pressure in the gaseous line is slightly increased in order to grow the liquid puddle. Time $t=0$ refers to the moment, when first liquid is visible to leave the needle.

For puddle formation using the developed device, the vertical needle tip is positioned slightly above a warm brass substrate. As a result the liquid puddle is still in contact with the needle tip during its growth, which finally fixes its position on the substrate below the needle. The growth of such a needle-bound liquid nitrogen puddle is shown as an example in Fig. 9. The needle, which is significantly tainted with frost, is placed perpendicularly close above the brass substrate during equilibrium operation of the device with $p_l \approx 1.1$ bar and the $p_g \approx 0.5$ bar. In order to grow the nitrogen puddle, the pressure in the outer tube is slightly increased resulting in a small excess mass flow of liquid nitrogen in the inner tube which finally causes liquid to leave the needle. Similar to the case of droplet formation shown in Fig. 8, first liquid nitrogen wets the outside of the needle and in the shown case of puddle formation it even reaches up to the rubber tube which is imposed on the needle as described before. As a result the entire needle tip is wetted and consequently immersed into the growing puddle; a liquid neck forms below the rubber tube during puddle growth. Compared to the extreme mobility of an unbounded nitrogen Leidenfrost droplet on a warm surface, bounding of the nitrogen puddle to the suspending needle drastically increases the controllability of the liquid and thus, allows detailed examination of the situation and the involved processes such as for the situation with a non-cryogenic liquid^22^.

## Conclusion

An original design approach for a lab-device for the supply of pure liquid nitrogen from a hypodermic stainless steel needle has been presented. The device allows supply of liquid nitrogen without contamination with its own vapor and variation of its operational conditions enables adjustment of the resulting mass flow of liquid nitrogen leaving the needle, which finally offers different possible applications of the device within a lab-environment such as e.g. droplet or liquid jet generation.

The device design and its operation have been described in detail and the device has been quantitatively characterized for continuous operation during the supply of a free liquid jet. In addition to the detailed characterization for jet generation, the device versatility has been briefly demonstrated based on the generation of a liquid nitrogen droplet and a needle-bound liquid puddle on a warm substrate. The device allows flexible handling of small portions of liquid nitrogen and compared to currently existing approaches it enables detailed examination of physical processes associated with liquid nitrogen in a significantly better controlled way. In its current state, the device is still accompanied by some remaining teething troubles. For example, oscillatory behavior during equilibrium device operation, i.e. without intending an outflow from the needle tip, complicates finding proper operational conditions for droplet generation or puddle formation. Nevertheless, already in its current development state, the advantages associated with the device in terms of its flexibility and the controllability of liquid nitrogen handling prevail compared to the problems still associated with the approach.

In the present work, only the relevant pressures in the liquid and gaseous domain have been varied. However, numerous other parameters such as e.g. the number and size of the orifices in the inner needle or the sizes of the inner and outer needle, may affect device operation and thus, presumably also its stability and reliability. Although the device already now drastically extends the possibilities for liquid nitrogen handling for fundamental research, significant potential for improvement of the device functionality is expected. For that purpose the exhaustive descriptions and characterization provided in the present work allow reproduction of the device in other laboratories, comprising its detailed examination and further optimization.

While in the present study the device has been only used and characterized for liquid nitrogen, the methodology may be theoretically also used for other cryogenic liquids. As long as sacrificial evaporation in the outer tube is sufficient to compensate for the heat transfer from the ambient to the system, the method may generally work as intended. However, unfavorable fluid properties such as a comparably low latent heat of evaporation combined with a low saturation temperature of the liquid may require impractically high mass flow rates for sufficient sacrificial evaporation. Besides a significantly lower device efficiency, the required mass flow rates presumably may even prevent a compact device design such as developed in the present study.

So far, the working principle of the device has been only elucidated from the perspective to use it for fundamental research with the supplied liquid. However, both the basic principle of sacrificial evaporation of a portion of the supplied liquid or even the entire design approach may be also exploited for other technical or scientific applications. Concluding, the presented approach represents both a promising solution for taming cryogenic liquids for laboratory purposes, and a promising building block for the application of the presented principle in other technical systems.

## Equipment and settings

Figure 1 has been generated using Inkscape 0.92.3; Fig. 2 has been exported from Solidworks 2020 and colored using Microsoft Paint; and in Figs. 3, 4, 8 and 9 images from high-speed videos are shown which have been stamped using Matlab R2020b.

## Supplementary Information

Supplementary Information.

## Data Availability

All data generated or analysed during this study are included in this published article. A detailed description of the device operation and its different operation modes can be found in the supplementary information for the present manuscript. Further data such as the high-speed videos being the base for a part of the data presented in the current work are available from the corresponding author on reasonable request.
