# Supplementary material for: Development of a lab-device for evaporation-free supply of pure liquid nitrogen for droplet- and jet-generation
Source: Sci Rep. 2023 Apr 19;13:6421. doi: 10.1038/s41598-023-31955-4 (PMC10115872; doi:10.1038/s41598-023-31955-4)
Supplement: Supplementary file 1 — Supplementary Information. [file 41598_2023_31955_MOESM1_ESM.pdf]

Supplementary Information for: Taming the Untamable: Towards a Lab-Device for Evaporation-free Supply of Pure Liquid Nitrogen for Droplet- and Jet-Generation

Markus Schremb[1,*], Marijn Kalter[1], and Srinivas Vanapalli[1,]**

[1]Applied Thermal Sciences Lab; Energy, Materials and Systems cluster; Faculty of Science and Technology; University of Twente; Postbus 217, 7500AE Enschede, The Netherlands
[*]m.schremb@utwente.nl, **s.vanapalli@utwente.nl

Operation Modes

Extending on the brief and simplified description of device operation in the main manuscript, the general working principle, device operation for the generation of individual droplets or a continuous jet of liquid nitrogen, as well as the procedure for device start-up are separately described in the following.

Equilibrium Operation (idle)

Since the start-up procedure for the device is significantly easier to understand after understanding the general working principle of the device, it is convenient to start with explaining the latter. At the equilibrium operation point, the mass flow of liquid nitrogen through the inner tube equals the mass flow of gaseous nitrogen through the outer tube. The working pressures in the inner and outer tube depend on each other in order to achieve equilibrium operation, but are in principle variable. They control the inflow of sacrificial liquid nitrogen into the outer tube and thus, they finally control the size of the heat sink through sacrificial evaporation. The lower the pressure in the outer tube (and thus, the higher the liquid supply pressure), the more liquid is soaked into the outer tube and the lower is the saturation temperature in the outer tube and thus, the more heat can be dissipated. Due to non-avoidable evaporation of liquid nitrogen at the very end of the inner needle, i.e. between the orifices and the needle outlet, this part of the needle is typically dry during equilibrium operation and does not contain liquid nitrogen which actually ranges only up to the orifices.

If the mass flow of liquid nitrogen is too low compared to the equilibrium operation point, the orifices in the inner needle run dry such that liquid nitrogen is not anymore soaked into the outer tube. Instead, a mixture of nitrogen already evaporated inside the inner tube and (warm) ambient air is soaked through the orifices into the outer tube. In that case, the cooling effect from liquid evaporation in the outer tube is lost after the last portion of liquid nitrogen left in the outer tube is evaporated and the thermal equilibrium of the operating system collapses. Moreover, the inner needle may get blocked as a result of frost formation resulting from the inflow of wet ambient air. However, it is worth to note that the liquid inside the inner needle presumably does never fully cover the orifices and thus, also during equilibrium operation of the device a certain amount of ambient air may always be soaked through the needle tip. If the device is operated in a wet ambient, this effect may always lead to eventual blocking of the inner needle, even if the mass flows in the inner and outer needle are equal.

Compared to the relative fast response of the thermodynamic processes on operational changes of the device, the large thermal mass of its components results in a certain thermal inertia of the entire system. As a consequence, the component temperatures do not change instantaneously after a change of the operational conditions of the device, equipping the device with a certain robustness against operational changes around the equilibrium operation point. However, after some time of operation without proper inflow and evaporation of liquid nitrogen in the outer tube, the thermal equilibrium of the system may collapse, resulting in a warm-up of the system finally leading to evaporation inside the inner tube, such as shown in Figure 4 in the manuscript.

During equilibrium operation, i.e. without an outflow at the needle tip, the orifices may be temporarily (partially) dry. In that case, the mass flow from the inner to the outer tube is reduced and less liquid may expand in the outer tube compared to the case with completely wetted orifices. As a result, the pressure in the outer tube is reduced which is associated with a lower saturation temperature, thus theoretically enabling a larger heat flux from the inner to the outer tube, which is primarily proportional to the

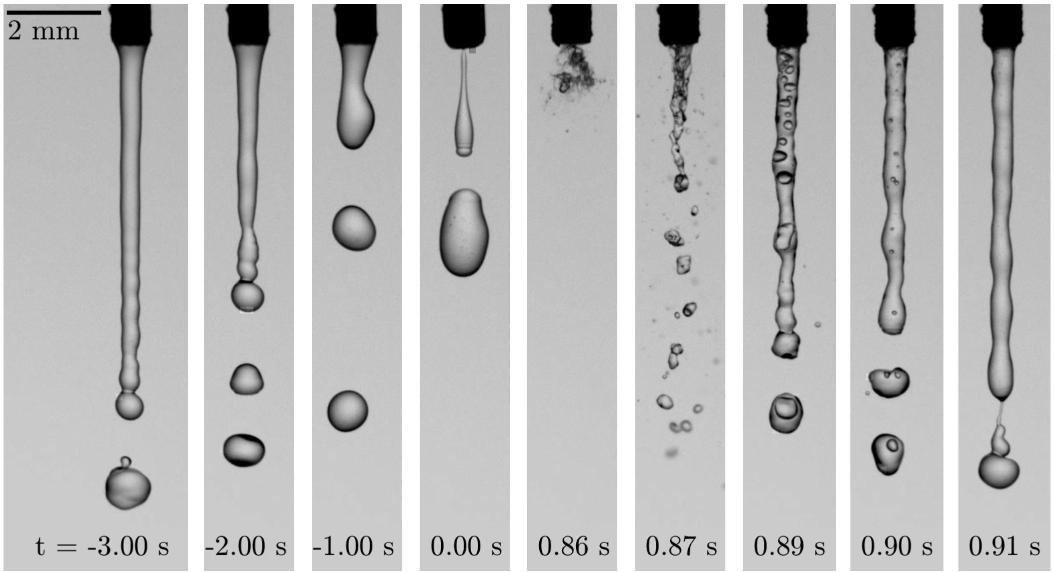

Figure S1. Unstable behavior of the device operated close to equilibrium at $p_l \approx 1.1\,\text{bar}$ and $p_g \approx 0.7\,\text{bar}$. Time $t = 0$ refers to the moment, when the last liquid leaves the needle before liquid supply is temporarily completely interrupted until $t = 0.86\,\text{s}$. Note the varying time intervals for $t < 0$ and $t > 0$.

temperature difference between them. However, at the same time a lower liquid mass flux into the outer tube is associated with a smaller theoretical heat sink size in the form of nitrogen evaporation in the outer tube. These effects are actually counteracting and drastically complicate finding stable conditions for equilibrium operation. Unstable behavior of the device for conditions close to equilibrium operation with $p_l \approx 1.1\,\text{bar}$ and $p_g \approx 0.7\,\text{bar}$ is shown for example in Figure S1. For a pure liquid jet and thus completely wetted orifices ($t < 0$) the outflow rate from the needle continuously decreases, which is associated with a decreasing jet break-up length. At some moment, $t = 0$, the last liquid leaves the needle and for a certain period the outflow completely stagnates. During that time the pressure in the outer tube decreases (increasing cooling effect) and so does the mass flow into it (decreasing cooling effect), while a portion of liquid may remain in the outer needle. The increased cooling effect due to a reduced pressure seemingly dominates the situation compared to the decreased cooling due to a lower mass flow into the outer tube and as a result, after some time of stagnating outflow, at $t = 0.86\,\text{s}$ liquid nitrogen accompanied by a significant amount of gaseous nitrogen explosively leaves the needle again. Due to the thermal inertia of the system increased cooling from dry orifices continues stabilizing the liquid jet, finally resulting in a pure liquid jet without any gas inclusions again at $t = 0.91\,\text{s}$. For completely wetted orifices, the pressure in the outer tube increases again and the described cycle repeats, finally resulting in oscillation of the device operation between a pure liquid and a stagnating outflow. While the effect of the wetting state at the orifices on the mass flow and pressure in the outer tube are actually experimental observations, the subsequent interpretation of their effect on the oscillatory behavior including device re-stabilization is only hypothesized. Actually, also pressure fluctuations in the liquid and gaseous domain can not be completely ruled out to contribute to the unstable device behavior. However, these pressure fluctuations in the system may be also a result of the oscillatory behavior. Therefore, based on the present data the primary reason for the observed behavior can not be certainly clarified and requires further examination.

Note that the described oscillatory behavior is not only relevant for equilibrium device operation but also for jet generation with a minimum of cooling from the outer tube, i.e. with a rather high pressure in the outer tube, as shown in Figure S2.

Jet Generation

If the mass flow of liquid nitrogen is increased compared to the equilibrium operation point by increasing the dewar pressure or increasing the pressure in the outer tube, liquid leaves the inner needle. For generating a liquid jet at the needle outlet the pressure inside the dewar is set to a constant value above the pressure associated with equilibrium operation. For a constant pressure in the outer tube, increasing the liquid pressure results in an increased outflow rate and thus, increased jet velocity. Also an increase of the pressure in the outer tube actually results in an excess of liquid nitrogen flow through the inner tube and thus, liquid nitrogen leaving the inner needle outlet. However, in that case also the cooling effect of evaporation in the outer tube is decreased, eventually making the system thermally less stable, as discussed in the context of Figure 6 in the main

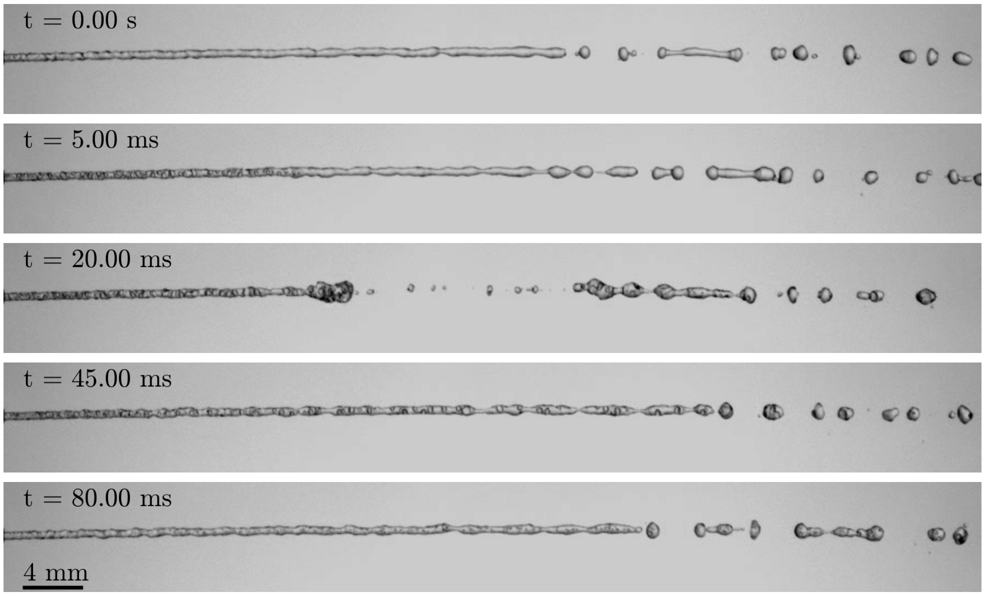

Figure S2. Oscillatory device operation for the case of slightly too little cooling from sacrificial evaporation during continuous operation of the device at $p_l \approx 1.8$ bar and $p_g \approx 0.82$ bar. The outflow from the needle alternates between a pure liquid jet and a liquid jet with contaminating gas bubbles. Time $t = 0$ refers to the first appearance of gas bubbles after a phase of pure liquid leaving the needle.

manuscript. In that case, evaporation in the inner tube is eventually not completely prevented and a multiphase mixture leaves the inner needle.

As already mentioned in the manuscript, the device may show unstable oscillatory behavior also during jet generation. Too little cooling from liquid evaporation in the outer tube may cause liquid evaporation in the inner tube, such that the orifices may be only partially wetted with liquid nitrogen. As a result the pressure and saturation temperature in the outer tube decrease finally increasing the cooling effect again. It suppresses evaporation in the inner tube such that the orifices become completely wetted again which is associated with an increasing pressure in the outer tube, causing a slightly too little cooling effect again. As a result the outflow from the needle finally oscillates between a pure liquid jet and a liquid jet containing a certain amount of nitrogen evaporated in the inner tube, which is shown as an example in Figure S2. A jet contaminated with gas bubbles can be clearly distinguished from a pure liquid jet due to its darker appearance. As shown in the figure, very similar to the explosive exit of liquid and gaseous nitrogen during equilibrium operation shown in Figure S1, also during transition from a pure liquid jet to a jet contaminated with gas bubbles ($t > 0$), a multiphase mixture may explosively leave the needle such as shown for $t = 20$ ms in Figure S2. However, after transition between the two states the multiphase jet at the needle outlet becomes homogeneous as shown for $t = 45$ ms. The gas bubbles in the jet decrease both the mass flow rate and the resulting pressure in the outer tube. As a result the cooling effect increases, finally re-stabilizing the jet to become purely liquid again. Although the gas content in the exiting jet is already significantly reduced at $t = 80$ ms, it actually takes until $t \approx 3.6$ s before no gas bubbles are anymore visible in the jet for the given conditions. Interestingly, that time well coincides with the time at which first gas bubbles become visible in the exiting jet after interruption of the connection to the vacuum pump, as discussed in the context of Figure 4 of the manuscript.

Droplet Generation

In order to generate an individual droplet leaving the needle outlet, comparable to common drop-on-demand approaches for non-cryogenic liquids, the pressure inside the outer tube is temporarily increased above its pressure at equilibrium operation. For that purpose a time controlled solenoid valve with a minimum opening time of approximately 50 ms is used to temporarily connect the dewar lid connection used for common depressurization of the outer tube to a source providing gaseous nitrogen at an elevated pressure above the set dewar pressure. By this, depending on the opening time of the valve and the set pressure in the over-pressure source, a certain amount of liquid is forced out of the inner needle and finally forms a droplet. Common approaches for drop-on-demand droplet generators based on pressurization of the liquid usually work with an impulse length below 1 ms applied to the liquid reservoir which is commonly placed close to the position of droplet formation. However, in

the present device the distance between the source for temporary (over-)pressurization and the position of droplet generation at the needle outlet results in a significant damping of the applied pressure peak. As a result, the liquid droplet at the needle outlet is rather formed as a consequence of a continuous overflow of the liquid in the inner tube than as the result of liquid dynamically pushed through the needle exit due to a pressure wave.

The previous descriptions represent the idealised/simplified theory of the device operation, which actually involves many physical processes such as the throttling flow of a liquid through an orifice, multiphase flow through rough-walled tubes, nucleation, liquid boiling/evaporation, transient cool down of the inner needle tip during droplet generation, etc. Since all of these processes are associated with certain stochastics, finally also the resulting device operation is accompanied by certain stochastics and non-avoidable fluctuations. While the device operation for the generation of a liquid jet is rather insensitive against these stochastics in terms of the resulting characteristic quantities such as jet velocity and device efficiency, they particularly affect droplet generation which is accompanied by a decreased repeatability of the process. The part of the inner needle usually dry during equilibrium operation, i.e. the 7 mm at the end of the needle, represents a volume of approximately 1.4 µl while the typical volume flow of liquid nitrogen during equilibrium operation is in the order ($\mathcal{O}(100\,\mu l/s)$). As a result, in particular, the equilibrium operation point is very sensitive towards fluctuations of the operational conditions and thus, rather unstable. Consequently, the resulting droplet diameter is not well controllable at the moment and may vary even without changing the operational conditions of the device; or even no droplet is formed without an obvious reason.

Nevertheless, compared to previous approaches for the design of a droplet generator for cryogenic liquids which are associated with a significant inflexibility of the device, the developed method is still advantages since it allows handling of the resulting jet and droplets as if they are from a non-cryogenic liquid; even if droplet formation is associated with a certain unpredictability.

Device Start-up

As already implied through the previous explanations, cooling down the device using the settings applied for normal device operation does not work. Instead, for the start-up of the device, i.e. for cooling down of the flexible tubes and all relevant components to a temperature at which the evaporation rate in the inner tube is negligible, the needle tip is submerged into an open bath of liquid nitrogen while depressurizing the outer tube. In that case, liquid nitrogen from the open bath may directly enter into the inner needle in order to flow through the orifices into the outer needle/tube from where it cools down all device components through the same process as during normal device operation. In order to avoid soaking in of ice crystals or other contaminating particles from the open nitrogen bath, a fine mesh filter element is attached to the needle tip during that procedure. Before the tubes are not yet sufficiently cooled down to minimize evaporation in the inner tube, pressurizing the dewar is actually counteracting the cool-down process. In that case evaporation of liquid entering the inner tube from the dewar reduces the amount of liquid soaked into the outer tube and eventually even leads to nitrogen gas flowing out of the inner needle into the open nitrogen bath, which significantly hinders or even fully prevents soaking in of liquid nitrogen through the orifices from the bath. In order to prevent that effect and to reduce the cool-down time of the device, pressure build-up in the dewar is avoided by opening the venting valve at the dewar during the described device cool-down. After sufficient pre-cooling of the device via that method, a temperature of the inner tube below or close to the saturation temperature at intended liquid pressure ($\approx 100 - 110\,\mathrm{K}$ turned out to be low enough) allows to close the venting valve in order to pressurize the dewar. As a result, the cooled inner tube fills with liquid nitrogen from the dewar without too much further liquid evaporation in the inner tube. After the pre-cooled inner tube is completely filled with liquid nitrogen from the dewar, the needle tip can be removed from the liquid nitrogen bath and the device may be operated as described in the previous sections, i.e. based on the flow of a portion of the liquid in the inner tube through the orifices and its sacrificial evaporation in the outer tube.

Starting with a filled and thermally settled dewar, and the remaining system being at room temperature, the described start-up procedure takes approximately $20 - 25$ minutes until the device may be used in the intended way. Depending on its actual operational conditions, the device may be used for approximately 45 minutes when starting with a completely filled dewar.
